# Efficacy and safety of anlotinib hydrochloride combined with concurrent radiotherapy in the treatment of locally advanced cervical cancer: a single-arm, single-center, exploratory, phase II clinical study

**DOI:** 10.3389/fonc.2025.1662160

**Published:** 2025-11-20

**Authors:** Hongfei Liu, Xuezhi Chang, Ye Hong, Hao Yin, Haiyan Zhang, Gulizhaer Wufuer, Shabiremu Abuduaini, Yali Jiang

**Affiliations:** 1 Tumor Radiotherapy Department, Ili Kazakh Autonomous Prefecture State Friendship Hospital, Yining, China; 2 Ili & Jiangsu Joint Institute of Health, Ili Kazakh Autonomous Prefecture State Friendship Hospital, Yining, China

**Keywords:** locally advanced cervical cancer, anlotinib, radiotherapy, efficacy, safety, progression-free survival

## Abstract

**Objective:**

This study aims to evaluate the therapeutic efficacy and safety of anlotinib, a multitarget tyrosine kinase inhibitor, combined with radiotherapy in patients with locally advanced cervical cancer (LACC).

**Methods:**

A prospective single-center study enrolled 62 eligible LACC patients (intention-to-treat [ITT] population) between May 2023 and January 2024, with 53 completing the full treatment course (per-protocol [PP] population). Patients received anlotinib (10 mg/day, days 1–14, 21-day cycles) combined with intensity-modulated radiotherapy (IMRT) and intracavitary brachytherapy. Efficacy was assessed using RECIST v1.1 criteria, including objective response rate (ORR), disease control rate (DCR), and progression-free survival (PFS). Safety was evaluated by monitoring adverse events. Cox regression analyses identified factors influencing PFS, with subgroup analyses by FIGO stage (I–III vs. IV).

**Results:**

In the PP population, ORR was 41.51% (5.66% complete response [CR], 35.85% partial response [PR]), and DCR was 83.02%. The ITT population showed lower ORR (35.48%) and DCR (70.97%). Common adverse events included fatigue (28.30%), hypothyroidism (22.64%), and diarrhea (22.64%), with manageable severity. Cox regression revealed that age, diabetes, hypertension, cancer history, and metastatic status significantly influenced PFS. Subgroup analyses showed no statistical differences in efficacy (ORR, DCR) between Stage I–III and IV patients, though Stage IV patients experienced earlier progression.

**Conclusion:**

Anlotinib combined with radiotherapy demonstrates promising efficacy and acceptable safety in LACC, with a favorable DCR. The multi-target mechanism of anlotinib may contribute to consistent efficacy across different FIGO stages, supporting its potential as a therapeutic option for LACC. Larger-scale trials are warranted to validate these findings.

## 1 Introduction

The spectrum of gynecological malignancies—cervical, endometrial, and ovarian cancers—poses significant global health burdens, with cervical cancer (CC) representing the second highest incidence and mortality rate in resource-limited settings (1). With more than 1 million new cases diagnosed annually, CC claims nearly 300,000 lives each year across the globe (2). In China, the incidence of CC has been rising steadily over the years (3). Although the widespread adoption of CC screening has led to a recent decline in incidence rates, it remains a significant public health burden (4). Since the early symptoms of CC often resemble those of other gynecological conditions, many patients are diagnosed at a locally advanced stage (5). Compared to early-stage CC, locally advanced cervical cancer (LACC) presents greater therapeutic challenges (6). Approximately 30% of LACC patients experience recurrence within 5 years, with rapid disease progression post-recurrence (7). The 5-year survival rate for recurrent LACC drops to less than 10% (7). Consequently, oncologists are committed to developing more intensive and effective treatment strategies for this high-risk patient population.

For patients with LACC, radiotherapy is the standard clinical treatment for LACC (8). However, elderly LACC patients with comorbidities often exhibit poor tolerance to radiotherapy alone, frequently resulting in suboptimal treatment outcomes (9). Compared to radiotherapy alone, cisplatin-based concurrent chemoradiotherapy significantly improves the 5-year survival rate in LACC patients, demonstrating efficacy even in elderly populations (10). However, approximately 50% of patients develop myelosuppression, and 20% are unable to complete the planned treatment course due to toxicity (10). In recent years, the incorporation of angiogenesis inhibitors into radiotherapy for LACC has been extensively investigated, with these agents demonstrating promising radiosensitizing effects (11). Building on this evidence, exploring combination regimens of angiogenesis inhibitors with radiotherapy has emerged as a pivotal strategy for controlling tumor progression in LACC patients.

Anlotinib is a novel multitarget tyrosine kinase inhibitor (TKI) that potently suppresses tumor angiogenesis and impedes cancer progression (12). Previous studies have confirmed that anlotinib hydrochloride inhibits phosphorylation of target proteins in a dose-dependent manner and induces tumor cell apoptosis (13). Clinical trials demonstrate its anti-tumor efficacy and favorable safety profile in treating various malignancies, including non-small cell lung cancer (NSCLC) and colorectal cancer (CRC) (14, 15). Although anlotinib hydrochloride has demonstrated antitumor activity in other malignancies, its efficacy in LACC remains unclear (16). This study aims to evaluate the therapeutic potential of anlotinib combined with concurrent radiotherapy in LACC, providing preliminary data for future clinical investigations and expanding treatment options for gynecologic oncology patients.

## 2 Material and methods

### 2.1 Patients

This study utilized PASS 15.0 software and adopted a single-arm design with the primary endpoint being the objective response rate (ORR). Based on historical data, the standard LACC treatment yields an ORR of approximately 20%. Assuming that anlotinib combined with radiotherapy can increase the ORR to 40%, with a significance level of α=0.05 (two-sided) and a test power of 1-β=0.80, the analysis determined that a minimum sample size of 36 patients is required. Considering a 20% dropout rate, the final target enrollment was set at 44 participants.

This prospective study enrolled 78 CC patients at Yili Prefecture Friendship Hospital between May 2023 and January 2024. After exclusion of 9 cases not meeting LACC criteria and 3 cases with vital organ metastases, 66 subjects eventually entered the safety lead-in study. Among them, 3 withdrew due to safety concerns and 1 discontinued because of an adverse event. Ultimately, leaving 62 eligible participants, they were intention-to-treat (ITT) population. During the treatment period, 5 patients were non-adherent to the medication regimen. 4 patients were lost to follow-up due to personal reasons and were automatically withdrawn from the study. The final cohort comprised 53 patients who completed protocol-defined radiotherapy, they were per protocol (PP) population, undergoing imaging assessments every 8 weeks with 100% retention. The study cutoff time was defined as ≥12 months after the enrollment date of the last participant. This sample size met the pre-specified minimum requirement (calculated n=53). Patient selection flow is detailed in **Supplementary Figure 1**. Patients were stratified into Stage I-III and Stage IV groups according to the International Federation of Gynecology and Obstetrics (FIGO) staging system, followed by subgroup analyses to identify factors associated with progression-free survival (PFS) in LACC patients. The trial was conducted in compliance with the Declaration of Helsinki and ICH-GCP guidelines, with written informed consent obtained from all participants. Ethical approval was granted by Yili Friendship Hospital’s Institutional Review Board (Approval No.: BMR2022-04).This study has been registered in the Chinese Clinical Trial Registry (https://www.chictr.org.cn/), with the registration number ChiCTR2200062885.

Inclusion criteria: (1) Voluntary participation with written informed consent. (2) Age 18–75 years. (3) Meeting the diagnostic criteria of NCCN Cervical Cancer Guidelines 2020 (17), with cervical squamous cell carcinoma or adenocarcinoma confirmed as CC, and LACC confirmed by cervical cytology. (4) Clinical stage IB3, IIA2, or IIB-IVA. (5) No prior radiotherapy, chemotherapy, molecular targeted therapy, immunotherapy, or other antitumor treatments. (6) Measurable primary tumor. (7) No vital organ metastases. (8) Karnofsky Performance Status (KPS) ≥70. (9) Life expectancy ≥3 months. (10) Women of childbearing potential must use contraception during the study period. (11) Good compliance with treatment and follow-up.

Exclusion criteria: (1) Evidence of vital organ metastases. (2) Prior surgical treatment of the primary tumor or lymph nodes (except biopsy). (3) Previous radiotherapy, chemotherapy, or molecular targeted therapy for the primary tumor or lymph nodes. (4) Diagnosis of other malignancies within 5 years before study initiation. (5) Participation in other drug trials within the past month. (6) Pregnant or lactating women, or women who refuse contraception during the treatment observation period. (7) History of severe allergies or specific hypersensitivity. (8) History of severe pulmonary or cardiac diseases. (9) Refusal or inability to sign the informed consent for trial participation. (10) Drug abuse or alcohol addiction. (11) Personality or psychiatric disorders, or individuals with no or limited legal capacity.

Termination and withdrawal criteria: (1) Patients who did not adhere to the prescribed medication or failed to complete the treatment plan during the trial period. (2) Violation of the study protocol requirements. (3) Poor-quality data records, incomplete or inaccurate information. (4) Failure to complete the full course of radiotherapy. (5) Loss of contact with the patient.

### 2.2 Safety lead-in and dose determination

Prior to the formal enrollment of the phase II cohort, a safety lead-in was conducted patients (n=66) to determine the tolerated dose of anlotinib in combination with concurrent radiotherapy, starting with an 8 mg dose of anlotinib. The primary observation indicator is the occurrence of acute abnormal uterine bleeding (AUB) during radiotherapy and drug treatment, defined as severe bleeding that requires urgent intervention by the physician to prevent further blood loss (18). If no acute AUB occurs, the dose of anlotinib may be appropriately increased. If one patient experiences acute AUB, the study will be immediately terminated, as the combination of radiotherapy and 12 mg anlotinib is deemed unsafe. During the trial, one patient withdrew due to acute AUB, and three patients withdrew due to chronic AUB over safety concerns.

### 2.3 Treatment

Anlotinib was used in combination with radiotherapy. During radiotherapy, patients orally took Anlotinib Hydrochloride Capsules (Chia Tai Tianqing Pharmaceutical Group Co., Ltd., specification: 10 mg, National Drug Approval No. H20180003) at a dose of 10 mg once daily from day 1 to day 14, with 21 days constituting one cycle, and a planned administration of 2 cycles.

Radiotherapy was delivered using intensity-modulated radiotherapy (IMRT), with the equipment being the TrueBeam^®^ system from Varian Medical Systems in the United States. Patients were positioned in the prone position on a belly board, immobilized with a thermoplastic body mold. The radiotherapy target volumes included the gross tumor volume, para-aortic region, pelvic region, common iliac, internal iliac, and external iliac lymphatic drainage areas, clinical target volume, internal target volume, and planning target volume. The external irradiation field extended superiorly to the bifurcation of the abdominal aorta into the left and right common iliac arteries and inferiorly to the lower edge of the obturator foramen. The total radiotherapy dose was 50 Gy delivered in 25 fractions at 2 Gy per fraction, administered 5 times per week, a total of 5 weeks will be conducted. After delivering 40 Gy, a repeat contrast-enhanced pelvic CT was performed. If significant changes in the lesion extent were confirmed, the radiotherapy target volumes and dose were adjusted accordingly. No modifications were made to the radiotherapy target volumes or prescribed dose for any patient, and all patients (n=62) completed 100% of the planned external-beam radiotherapy dose. All patients received intracavitary brachytherapy within one week after completing external beam radiation therapy at our institution, undergoing high-dose-rate three-dimensional brachytherapy guided by CT (Siemens Healthineers) using Varian Brachytherapy Solutions (Varian Medical Systems). The prescribed dose was either 30 Gy in 5 fractions or 28 Gy in 4 fractions, administered once weekly. Intracavitary brachytherapy is initiated after completion of 40 Gy IMRT and delivered over 6–7 weeks. All 62 patients received 100% of the prescribed brachytherapy dose.

### 2.4 Assessment

The researchers performed ultrasound or MRI examinations according to the Response Evaluation Criteria in Solid Tumors (RECIST) (19). All patients underwent imaging evaluation before enrollment to identify target lesions and measure the sum of their longest diameters. During the first treatment cycle, tumor response was assessed weekly, followed by assessments every two weeks thereafter. Lesion responses were categorized as follows: Complete response (CR): Disappearance of all target lesions, no new lesions, normalization of tumor markers, maintained for 4 weeks. Partial response (PR): ≥30% decrease in the sum of the longest diameters of all (one or more) baseline target lesions, maintained for 4 weeks. Stable disease (SD): Shrinkage of the sum of the longest diameters of all baseline target lesions insufficient to qualify as PR, or growth insufficient to qualify as progressive disease (PD). PD: ≥20% increase in the sum of the longest diameters of the smallest recorded target lesions, or the appearance of one or more new lesions. CR and PR required radiological confirmation at least 4 weeks after initial assessment. Patients with initial radiological evidence of response continued treatment until confirmed in subsequent evaluations. The duration of treatment for all patients, as well as the time to achieve CR, PR, SD, or PD, was recorded. Target lesion status was assessed every 8 weeks, and adverse events during treatment were documented.

### 2.5 End points

The primary endpoint was the ORR, defined as the proportion of patients achieving investigator-confirmed CR or PR, calculated as ORR=(number of CR+PR cases)/total number of cases×100%. Secondary endpoints included progression-free survival (PFS, defined as the time from first injection to disease recurrence or death from any cause) and disease control rate (DCR, the proportion of patients achieving CR, PR or SD), calculated as DCR=(number of CR+PR+SD cases)/total number of cases×100%.

### 2.6 Survival follow up

After completion of treatment, all patients underwent planned radiographic evaluations every 8 weeks during the survival follow-up period to assess target lesions and detect new lesions, with tumor response evaluation continuing until study cutoff or disease recurrence.

### 2.7 Observation indicators

Baseline data included age, ethnicity, marital status, duration of symptoms, diabetes, hypertension, history of other cancers, age at menarche, menstrual cycle length, menopausal status, gravidity, parity, number of abortions, height, weight, body mass index (BMI), smoking history, and alcohol consumption history. Additionally, indicators such as pathological type, FIGO stage, Eastern Cooperative Oncology Group performance status (ECOG PS), metastatic status, number of metastatic sites, and target lesion size were documented.

Physical examination findings at baseline were recorded for all patients, including vaginal discharge volume, color, presence of odor, and vaginal involvement status.

Baseline hematological parameters were analyzed, including lymphocyte percentage (LY%), neutrophil percentage (NEUT%), white blood cell count (WBC), platelet count (PLT), red blood cell count (RBC), fasting blood glucose (FBG), carcinoembryonic antigen (CEA), carbohydrate antigen 724 (CA724), alpha-fetoprotein (AFP), carbohydrate antigen 199 (CA199), carbohydrate antigen 125 (CA125), cytokeratin 19 fragment 21.1 (Cyfra21.1), carbohydrate antigen 153 (CA153), and squamous cell carcinoma antigen (SCC).

Analyze medication adherence within the ITT population during the treatment period.

The efficacy endpoints of this study included CR, PR, SD, PD, ORR, and DCR. Treatment response was assessed and recorded between 4 and 16 weeks after treatment initiation.

All adverse events during treatment were documented, including hypothyroidism, elevated aspartate aminotransferase (AST), hypertension, diarrhea, hypertriglyceridemia, anemia, hypercholesterolemia, rash, gingival swelling/pain, oral ulcers, fatigue, bleeding, perforation, radiation enteritis, radiation cystitis, and irregular bleeding.

After study termination, PFS was calculated for all patients, and results were visualized. Cox regression analysis was performed to identify significant factors influencing PFS, and a forest plot was generated.

Further stratification was conducted based on LACC stage (Stage I-III as one group, Stage IV as another group). Comparisons were made between the two groups for the above indicators to evaluate the treatment efficacy of anlotinib hydrochloride combined with radiotherapy in patients with different stages of LACC.

### 2.8 Statistical analysis

Data processing and analysis were performed using SPSS 27.0 software (IBM Corporation, Armonk, NY), while data visualization was conducted using Prism 10.0 (GraphPad, San Diego, CA) and R version 3.4.1. Normality was assessed using the Shapiro-Wilk test. Normally distributed quantitative data were expressed as mean ± standard deviation ( $\bar{x}\pm s$) and analyzed using independent samples t-test for between-group comparisons. Non-normally distributed quantitative data were expressed as median (Q_25_, Q_75_) and analyzed using Mann-Whitney U test for between-group comparisons. Categorical data were expressed as number (percentage) [n (%)] and analyzed using *χ²* test. DFS curves were plotted using the Kaplan-Meier method. *P* < 0.05 was considered statistically significant.

## 3 Results

### 3.1 Baseline characteristics

The median age of the ITT population was 57 years. The Uyghur ethnic group accounted for 41.94%, and 70.97% were married. Most patients had no history of diabetes (82.26%), hypertension (69.35%), or other cancers (93.55%). Menstrual characteristics were all within normal ranges. Postmenopausal women accounted for 69.35%. The median number of pregnancies was 5, with a median of 3 deliveries and 1 abortion. Height and weight measures fell within normal ranges, while BMI was slightly elevated. Smoking and alcohol consumption were reported in 46.77% and 9.68%, respectively. Squamous cell carcinoma was identified in 88.71%, and FIGO stage II–III disease accounted for 67.75%. An ECOG PS score of 0 was observed in 56.45%, and 90.32% showed no evidence of metastasis. Moreover, 91.94% of patients had 0–1 metastatic sites, and the mean target lesion size was 4.52 cm. **Table 1**.

**Table 1:** Baseline characteristics (N = 62).

Characteristic	Patients
Age, years	57.00 (46.75, 67.25)
Ethnic group	
Han ethnic group	16 (25.81)
Uyghurs ethnic group	26 (41.94)
Kazak ethnic group	20 (32.26)
Marital status	
Married	44 (70.97)
Single	2 (3.23)
Divorced or widowed	16 (25.81)
Duration of symptoms, months	3.00 (1.00, 8.00)
Diabetes	
Yes	11 (17.74)
No	51 (82.26)
Hypertension	
Yes	19 (30.65)
No	43 (69.35)
History of other cancers	
Yes	4 (6.45)
No	58 (93.55)
Age at menarche, years	15.00 (13.00, 15.00)
Duration of menstruation, days	5.00 (5.00, 5.00)
Menstrual cycle length, days	28.00 (28.00, 28.00)
Menopausal status	
Yes	43 (69.35)
No	19 (30.65)
Gravidity, n	5.00 (3.00, 6.25)
Parity, n	3.00 (3.00, 5.00)
Number of abortions, n	1.00 (0.00, 2.00)
Height, m	1.57 ± 0.06
Weight, kg	61.53 ± 11.03
BMI, kg/m^2^	24.88 ± 3.89
Smoking history	
Yes	29 (46.77)
No	33 (53.23)
Alcohol consumption	
Yes	6 (9.68)
No	56 (90.32)
Pathological type	
Squamous cell carcinoma	55 (88.71)
Adenocarcinoma	7 (11.29)
FIGO staging	
I	1 (1.61)
II	23 (37.10)
III	19 (30.65)
IV	19 (30.65)
ECOG PS	
0	35 (56.45)
1	24 (38.71)
2	3 (4.84)
Metastatic status	
Yes	6 (9.68)
No	56 (90.32)
Number of metastatic sites	
0~1	57 (91.94)
2	5 (8.06)
Target lesion size, cm	4.52 ± 0.75

BMI, Body mass index; FIGO, International Federation of Gynecology and Obstetrics; ECOG, Eastern Cooperative Oncology Group.

### 3.2 Physical examination findings

The physical examination findings are presented 75.81% had a scanty amount of vaginal discharge, 64.52% exhibited white-colored vaginal discharge, 77.42% showed no abnormal odor, and 91.94% had no vaginal invasion. **Supplementary Table 1**.

### 3.3 Complete blood count results

The complete blood count results are presented that the median/mean values of LY%, NEUT%, WBC, PLT, FBG, CEA, CA724, AFP, CA199, CA125, Cyfra21.1, CA153, and SCC in the ITT population were all within normal ranges, while the RBC levels in patients were below the normal reference range of 3.8-5.1×10¹²/L. **Supplementary Table 2**.

### 3.4 Medication adherence

In the ITT cohort, 53 patients (85.48%) received 100% of the planned total anlotinib dose, 57 patients (91.94%) received at least 80% of the planned dose, and all 58 patients (93.55%) received at least 70% of the planned dose. **Table 2**. 4 patients requested to withdraw from the study due to personal reasons, having received only 30% of the planned dose.

**Table 2:** Medication adherence (N = 62).

Relative dose intensity	Number of patients
100%	53 (85.48)
80%	57 (91.94)
70%	58 (93.55)
30%	62 (100.00)

### 3.5 Adverse events in the PP population

A safety analysis was performed on the per-protocol (PP) population who completed the full treatment cycle (**Table 3**). The incidence rates of adverse events were as follows: hypothyroidism (22.64%), elevated aspartate aminotransferase (AST) (11.32%), hypertension (15.09%), diarrhea (22.64%), hypertriglyceridemia (5.66%), anemia (9.43%), hypercholesterolemia (3.77%), rash (5.66%), gingival swelling and pain (9.43%), oral ulcer (5.66%), fatigue (28.30%), radiation enteritis (5.66%), radiation cystitis (7.55%), and irregular bleeding (1.89%).

**Table 3:** Adverse events in the PP population (N = 53).

Characteristic	Patients
Hypothyroidism	12 (22.64)
Elevated AST	6 (11.32)
Hypertension	8 (15.09)
Diarrhea	12 (22.64)
Hypertriglyceridemia	3 (5.66)
Anemia	5 (9.43)
Hypercholesterolemia	2 (3.77)
Rash	3 (5.66)
Gingival swelling and pain	5 (9.43)
Oral ulcer	3 (5.66)
Fatigue	15 (28.30)
Radiation enteritis	3 (5.66)
Radiation cystitis	4 (7.55)
Irregular bleeding	1 (1.89)

AST, Aspartate aminotransferase.

### 3.6 Efficacy measures per RECIST v1.1 guidelines

In the PP population, the ORR was 41.51% (consisting of 5.66% CR and 35.85% PR), with SD observed in 41.51% and PD in 16.98% of patients, resulting in a DCR of 83.02%. For the ITT population, the ORR was 35.48% (comprising 4.84% CR and 30.65% PR), with SD in 35.48% and PD in 14.52% of patients, yielding a DCR of 70.97%. Additionally, 14.52% of ITT cases were not evaluable (**Table 4**).

**Table 4:** Efficacy measures per RECIST v1.1 guidelines.

Overall study endpoint	ITT population (n=62)	PP population (n=53)
Primary endpoint		
ORR	22 (35.48)	22 (41.51)
Secondary endpoint		
CR	3 (4.84)	3 (5.66)
PR	19 (30.65)	19 (35.85)
SD	22 (35.48)	22 (41.51)
PD	9 (14.52)	9 (16.98)
DCR	44 (70.97)	44 (83.02)
Not assessed	9 (14.52)	—

ORR, Objective response rate; CR, Complete response; PR, Partial response; SD, Stable disease; PD, Progressive disease; DCR, Disease control rate.

**Figure 1:**
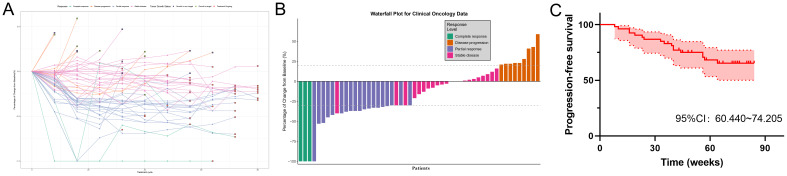
PP population efficacy assessment. **(A)** Spider plot of tumor diameter sum over time in the PP population. **(B)** Waterfall plot of treatment efficacy evaluation in the PP population. **(C)** PFS survival analysis in the PP population.

A comprehensive visualization of efficacy evaluation for all PP population patients is presented in **Figure 1**. The spider plot (**Figure 1A**) clearly demonstrates the therapeutic response dynamics of each patient over time. One CR patient showed recurrence of target lesions after initial complete remission, though without reaching PD criteria, with subsequent gradual improvement during continued treatment. Seven patients exhibited enlargement of target lesions, while nine developed new lesions. The waterfall plot analysis of PP population (**Figure 1B**) revealed, three confirmed CR cases, one patient achieved 100% disappearance of target lesions but failed to maintain this response for ≥4 weeks (classified as PR), three SD cases showed >30% reduction in target lesions without sustaining for 4 weeks, nine patients met PD criteria with >20% increase in target lesions. PFS outcomes for the PP population are shown in **Figure 1C**. Disease recurrence was observed starting from week 8, with the longest follow-up duration reaching 84 weeks. Notably, no mortality events occurred in the study population.

### 3.7 Univariate Cox proportional hazards regression analyses of PFS

**Figure 2:**
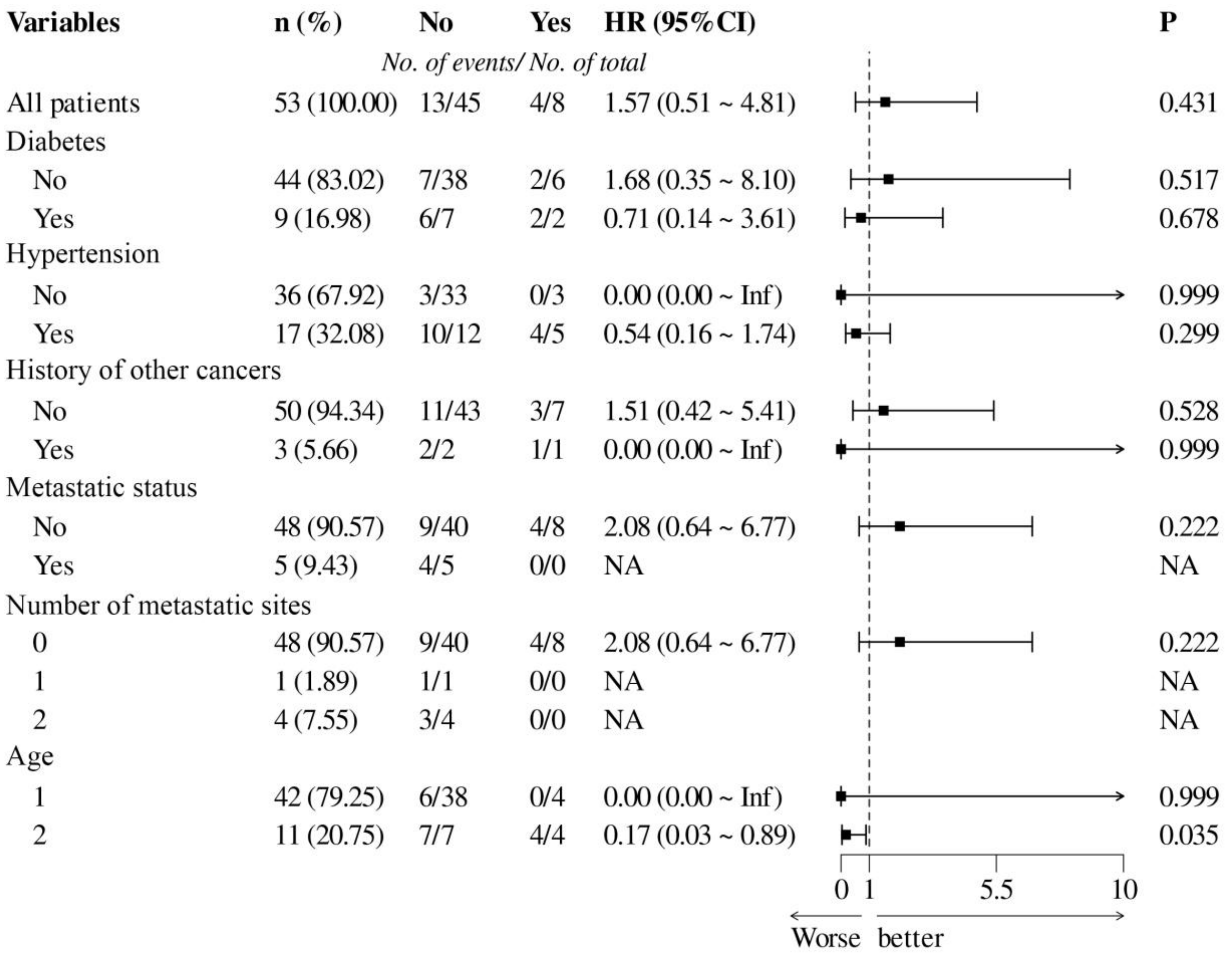
Forest plot of Cox regression analysis for PFS.

Age, diabetes, hypertension, cancer history, metastatic status, and number of metastatic sites showed statistically significant associations (*P* < 0.05) (**Table 5**). Significant predictors were visualized in the forest plot (**Figure 2**). Overall PFS outcomes were favorable in the study population. However, patients with diabetes or prior cancer history demonstrated worse PFS, those without metastasis (metastatic number=0) exhibited better PFS outcomes.

**Table 5:** Univariate Cox proportional hazards regression analyses of PFS.

Characteristic	Univariate		
	*HR*	95%CI	*P*
Age, years	1.135	1.064-1.212	<0.001
Ethnic group			
Han ethnic group			0.988
Uyghurs ethnic group	0.909	0.256-3.224	0.883
Kazak ethnic group	0.981	0.329-2.923	0.973
Marital status			
Married			0.982
Single	1.114	0.362-3.428	0.850
Divorced or widowed	0.000	0.000-9.999	0.985
Duration of symptoms, months	1.002	0.965-1.041	0.918
Diabetes	0.415	0.257-0.670	<0.001
Hypertension	0.240	0.128-0.450	<0.001
History of other cancers	0.312	0.163-0.599	<0.001
Age at menarche, years	1.152	0.787-1.686	0.466
Duration of menstruation, days	0.862	0.469-1.584	0.633
Menstrual cycle length, days	1.312	0.800-2.150	0.282
Menstrual blood loss, mL	0.953	0.870-1.044	0.301
Menopausal status	0.796	0.427-1.485	0.473
Gravidity, n	1.022	0.807-1.294	0.856
Parity, n	1.152	0.835-1.588	0.389
Number of abortions, n	0.882	0.578-1.345	0.559
Height, m	0.879	0.000-1639.382	0.973
Weight, kg	0.973	0.934-1.015	0.203
BMI, kg/m^2^	0.922	0.820-1.036	0.171
Smoking history	0.997	0.619-1.606	0.991
Alcohol consumption	0.956	0.457-2.001	0.906
Vaginal discharge amount			
Scanty			0.997
Moderate	9.999	0.000-19.958	0.947
Copious	9.999	0.000-19.958	0.947
Vaginal discharge color			
White			0.270
Purulent yellow	4.383	0.576-33.349	0.154
Blood-tinged	2.234	0.202-24.667	0.512
Presence of odor	1.317	0.378-4.584	0.665
Vaginal involvement	1.358	0.180-10.266	0.767
Pathological type	25.407	0.062-1043.354	0.292
FIGO staging			
I			0.221
II	18.634	0.000-186.590	0.941
III	29.649	0.000-296.790	0.938
IV	65.768	0.000-657.790	0.931
ECOG PS			
0			0.414
1	0.309	0.037-2.547	0.275
2	0.517	0.065-4.126	0.533
Metastatic status	0.421	0.240-0.741	0.003
Number of metastatic sites	2.676	1.445-4.955	0.002
Target lesion size, cm	0.873	0.452-1.686	0.686
LY%, %	1.033	0.980-1.089	0.226
NEUT%, %	0.998	0.956-1.042	0.918
WBC, 10^9^/L	0.859	0.680-1.084	0.200
PLT, 10^9^/L	0.743	0.356-1.552	0.430
RBC, 10^12^/L	0.999	0.995-1.004	0.767
FBG, mmol/L	0.679	0.413-1.118	0.128
CEA, ng/mL	0.998	0.977-1.019	0.818
CA724, U/mL	0.620	0.317-1.211	0.162
AFP, ng/mL	0.910	0.611-1.355	0.643
CA199, U/mL	0.985	0.942-1.031	0.523
CA125, U/mL	1.000	0.998-1.003	0.675
Cyfra21.1, ng/mL	1.009	0.992-1.026	0.294
CA153, U/mL	1.011	0.990-1.032	0.292
SCC, ng/mL	1.013	0.988-1.038	0.315
Hypothyroidism	0.792	0.469-1.335	0.381
Elevated AST	1.317	0.479-3.620	0.594
Hypertension	0.798	0.456-1.399	0.431
Diarrhea	0.806	0.477-1.361	0.419
Hypertriglyceridemia	4.750	0.080-282.980	0.455
Anemia	1.313	0.478-3.606	0.598
Hypercholesterolemia	0.648	0.236-1.783	0.401
Rash	1.047	0.381-2.876	0.929
Gingival swelling and pain	0.893	0.427-1.871	0.765
Oral ulcer	4.703	0.051-431.829	0.502
Fatigue	0.985	0.584-1.661	0.954
Radiation enteritis	1.047	0.381-2.876	0.929
Radiation cystitis	0.956	0.346-2.637	0.930
Irregular bleeding	4.572	0.005-433.564	0.664

### 3.8 Characteristics of patients by disease stage

The PP population was further stratified into Stage I-III and Stage IV groups based on FIGO staging. Statistical analysis revealed significant differences between the two groups in ethnic group and height (*P* < 0.05) (**Supplementary Table 3**). No significant differences were observed between the two groups in terms of physical examination findings, cervical cancer status, complete blood count parameters, or adverse event profiles (*P*>0.05) (**Supplementary Tables 4**-**7**).

### 3.9 Efficacy measures per RECIST v1.1 guidelines of patients by disease stage

The efficacy measures analysis stratified by disease stage is presented in **Supplementary Table 8**. No statistically significant differences were observed between the two patient groups for either the primary endpoint (ORR) or secondary endpoints (CR, PR, SD, PD, and DCR) (*P*>0.05).

**Figure 3:**
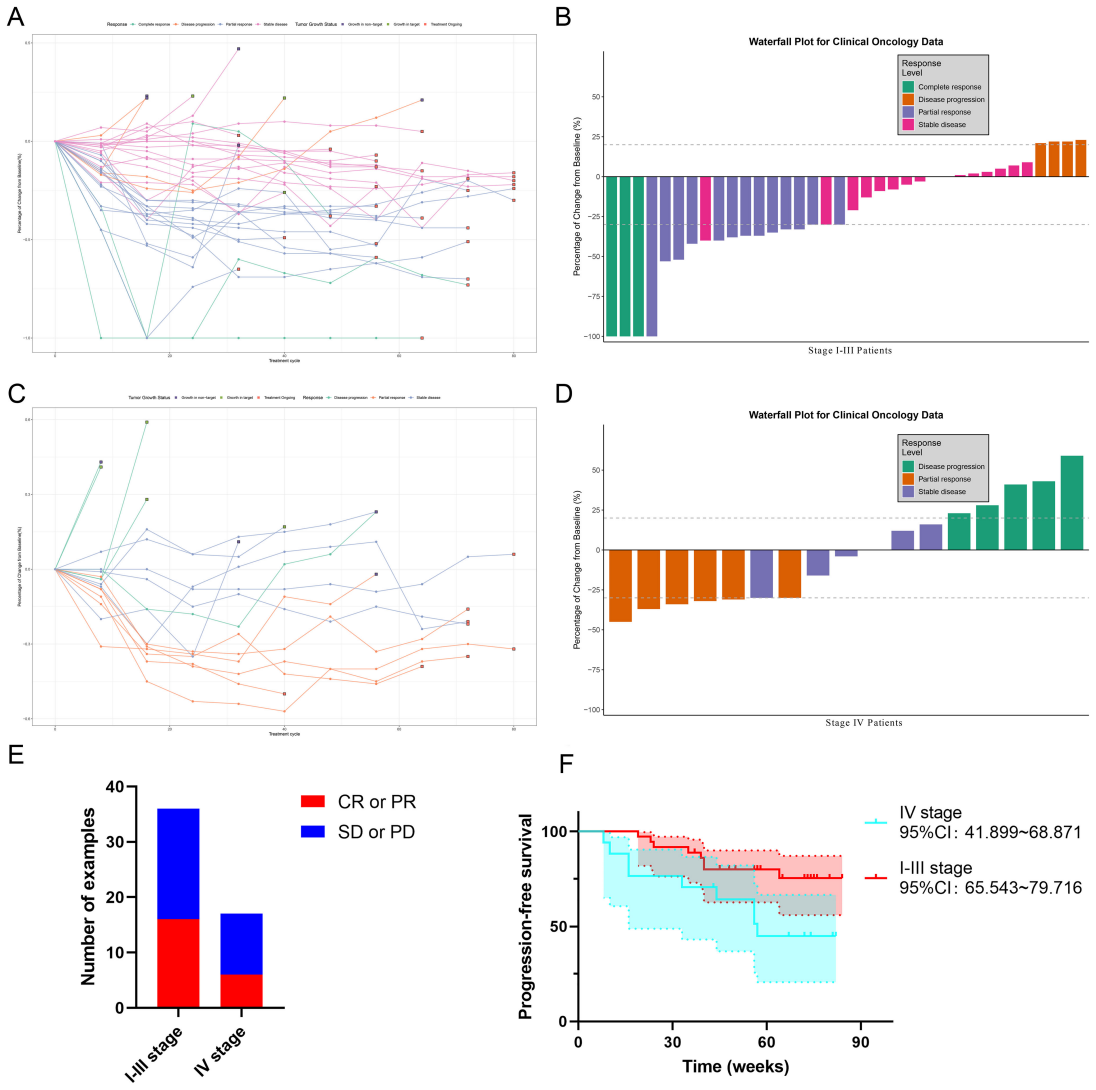
Efficacy-evaluable population efficacy assessment of patients by disease stage. **(A)** Spider plot of tumor diameter sum over time in the I-III population. **(B)** Waterfall plot of treatment efficacy evaluation in the I-III population. **(C)** Spider plot of tumor diameter sum over time in the IV population. **(D)** Waterfall plot of treatment efficacy evaluation in the IV population. **(E)** Efficacy assessment stratified according to disease staging. **(F)** Stratified analysis of PFS across different disease stages.

The visual analysis of efficacy evaluation for both patient groups is shown in **Figure 3**. The spider plot (**Figure 3A**) clearly displays the changes in treatment response over time for each Stage I-III patient. One CR patient experienced recurrence of target lesions after complete remission without meeting PD criteria, with subsequent gradual improvement during continued treatment. Three patients showed enlargement of target lesions, while five developed new lesions. The waterfall plot for Stage I-III patients (**Figure 3B**) shows three CR cases. One patient achieved 100% disappearance of target lesions but failed to maintain this response for ≥4 weeks, thus classified as PR. Two SD patients demonstrated >30% reduction in target lesions during treatment without sustaining for 4 weeks. Four patients showed >20% increase in target lesions, meeting PD criteria. For Stage IV patients, the spider plot (**Figure 3C**) reveals no CR cases, with four patients exhibiting target lesion enlargement and four developing new lesions. The Stage IV waterfall plot (**Figure 3D**) shows one SD patient with >30% reduction in target lesions that was not sustained for 4 weeks, and five PD patients with >20% increase in target lesions. The comparative efficacy visualization (**Figure 3E**) demonstrates that Stage IV patients had relatively fewer ORR cases. The PFS results by disease stage (**Figure 3F**) indicate that Stage IV patients experienced earlier progression events compared to Stage I-III patients, with no mortality occurring in either group.

### 3.10 Univariate Cox proportional hazards regression analysis of PFS in patients stratified by disease stage

The results of the COX regression analysis for PFS in Stage I-III patients are presented in **Supplementary Table 9**. Age, diabetes, hypertension, CA125, and CA153 showed statistically significant differences (*P* < 0.05). The forest plot visualization (**Supplementary Figure 2A**) demonstrates that patients without diabetes and with lower CA125 and CA153 levels had better PFS outcomes.

The results of the COX regression analysis for PFS in Stage IV patients are presented in **Supplementary Table 10**. Age, hypertension, cancer history, parity, metastatic status, and number of metastatic sites showed statistically significant differences (*P* < 0.05). The forest plot visualization (**Supplementary Figure 2B**) indicates that these significant factors did not demonstrate a pronounced impact on PFS outcomes in Stage IV patients.

## 4 Discussion

Anlotinib hydrochloride, as a novel multi-target tyrosine kinase inhibitor, has demonstrated significant antitumor activity and a favorable safety profile in the treatment of various malignancies (20). Its dual mechanism of action—inhibiting tumor angiogenesis and inducing tumor cell apoptosis—provides a new therapeutic strategy for cancer treatment (21). In this study, the efficacy and safety of anlotinib combined with radiotherapy in LACC were preliminarily validated, with its advantages reflected in a high DCR and a manageable adverse event profile (16). The results showed that the combination therapy achieved an ORR of 41.51% and a DCR of 83.02%, with most adverse events being mild to moderate and no severe or uncontrollable toxicities observed. These findings not only support the potential value of anlotinib in LACC treatment but also lay the groundwork for further exploration of its clinical applications in gynecological malignancies, providing additional evidence-based insights for optimizing therapeutic strategies in locally advanced cervical cancer.

In this study, nine patients in the ITT population experienced discomfort after using anlotinib hydrochloride, a phenomenon potentially associated with the drug’s multi-target mechanism of action and individual variability (22). As a multi-target tyrosine kinase inhibitor, anlotinib hydrochloride primarily exerts its antitumor effects by inhibiting signaling pathways such as vascular endothelial growth factor receptor (VEGFR), platelet-derived growth factor receptor (PDGFR), and fibroblast growth factor receptor (FGFR) (23). However, its potential impact on normal tissues may also lead to adverse reactions (24). VEGFR inhibition may cause vascular dysfunction, increasing the risk of hypertension or bleeding (25). Meanwhile, PDGFR and FGFR inhibition could impair tissue repair and metabolism, potentially triggering fatigue, oral ulcers, or thyroid dysfunction (26). Additionally, factors such as patient age, comorbidities, and the synergistic effects of combined radiotherapy may have further exacerbated these adverse reactions, leading to patient withdrawal from the study (27). Although most of these discomfort symptoms were mild to moderate and manageable, their occurrence highlights the need for close monitoring of patient responses in clinical practice, along with tailored treatment adjustments to optimize the balance between efficacy and safety (28). Future research could further explore biomarkers to predict patient tolerance to anlotinib hydrochloride, thereby providing a basis for personalized treatment.

The medication adherence results showed that over 85% of patients completed the full-dose treatment, indicating that the anlotinib regimen has a favorable safety profile. This finding is consistent with previous studies demonstrating a manageable toxicity spectrum (20). Particularly noteworthy is the maintained high dose completion rate despite the background of tissue damage commonly seen in cervical cancer patients following radiotherapy/chemotherapy, further supporting its applicability in real-world clinical scenarios (20). It is significant that only a small number of patients withdrew due to personal reasons, with no treatment discontinuations directly attributed to drug toxicity. This outcome compares favorably with clinical data from other anti-angiogenic agents. The demonstrated safety advantage of anlotinib provides strong support for its incorporation into combination therapy regimens for cervical cancer (23). Based on the excellent tolerability profile and reliable medication adherence observed in the current study, along with documented clinical activity evidence, anlotinib warrants further investigation through large-scale Phase III clinical trials to validate its efficacy in cervical cancer treatment. Subsequent studies should focus on combination strategies with immunotherapy or radiotherapy, potentially offering new therapeutic options for patients with advanced cervical cancer.

In the gynecological examination results of the ITT population, it was found that most patients did not have abnormal odors or vaginal involvement, and the vaginal discharge was mostly white and in small amounts. This may indicate that the vaginal environment in LACC patients is primarily characterized by low inflammatory response (29). Although most patients did not show obvious vaginal involvement, a small number of patients (8.06%) had vaginal invasion, suggesting that the local invasiveness of LACC tumors is heterogeneous (30). Moreover, the presence of bloody discharge and abnormal odors may reflect tumor necrosis or secondary infection (29). Although few patients in this study had these characteristics, they should still be taken seriously in clinical practice (30). Overall, the gynecological examination results reflect the local lesion characteristics of LACC patients and provide a certain reference for disease assessment and treatment monitoring.

In this study, the combination of anlotinib hydrochloride with radiotherapy for the treatment of LACC achieved an objective response rate of 41.51% (including 5.66% complete response and 35.85% partial response) and a disease control rate of 83.02% in the PP population. These results may be associated with the multi-target synergistic mechanism of anlotinib hydrochloride (25). As a tyrosine kinase inhibitor, it simultaneously suppresses VEGFR, PDGFR, and FGFR signaling pathways, effectively inhibiting tumor angiogenesis and disrupting stromal cell functions in the tumor microenvironment, thereby enhancing the local antitumor efficacy of radiotherapy (23). The study suggests that compared to other single-target anti-angiogenic drugs such as bevacizumab, anlotinib’s multi-target properties enable a more comprehensive inhibition of molecular pathways associated with tumor growth and metastasis (31). Additionally, the prolonged half-life of anlotinib hydrochloride ensures sustained target inhibition, and its potential to suppress post-radiotherapy DNA damage repair may contribute to synergistic effects (32). The randomized controlled study on anlotinib hydrochloride combined with radiotherapy demonstrated that anlotinib not only reduced distant metastasis but also significantly prolonged PFS in patients while decreasing the incidence of treatment-related adverse events (33). Preclinical studies have also shown that anlotinib can reverse tumor hypoxia, improving radiosensitivity (34). Furthermore, compared to other tyrosine kinase inhibitors (TKIs), anlotinib exhibits a more manageable safety profile, making it a clinically favorable option without compromising efficacy (23).

The results of this study show that LACC patients with diabetes or a history of cancer have a worse PFS, while those without metastasis have a better PFS. This may be attributed to the fact that hyperglycemia activates the insulin-like growth factor-1 (IGF-1) and PI3K/AKT/mTOR pro-survival signaling pathways, thereby enhancing the resistance of tumor cells to treatment (35). Meanwhile, the hyperglycemic microenvironment also promotes tumor angiogenesis and immune suppression, which weakens the effectiveness of anti-tumor treatment (36). For patients with a history of cancer, their tumor biology may be more aggressive, or there may be treatment-related selection of resistant clones, leading to an increased risk of disease progression (37). In contrast, the PFS advantage of patients without metastasis may be attributed to their lower tumor burden and the fact that the microenvironment has not yet developed extensive immune escape mechanisms (38). This allows the combination of Anlotinib Hydrochloride and radiotherapy to more effectively inhibit local lesions (39). Moreover, metastasis usually reflects that the tumor has acquired stronger invasive and metastatic capabilities, involving processes such as epithelial-mesenchymal transition (EMT) and stromal remodeling (27). These mechanisms may reduce the sensitivity to targeted therapy, thereby accelerating disease progression. Therefore, for LACC patients with a history of diabetes and cancer, in addition to more intensive blood glucose monitoring, physicians should exercise greater caution in determining radiotherapy dosage during treatment to minimize damage to surrounding tissues, thereby improving PFS and enhancing the patient’s quality of life.

In the comparison of gynecological examination results, this study found no statistically significant differences (*P*>0.05) in vaginal discharge volume, color, odor, or vaginal invasion between LACC patients at different stages (I-III vs. IV). This suggests that despite differences in tumor staging, the clinical manifestations of local lesions may be more influenced by common factors such as tumor biological behavior or microenvironment rather than staging variations (40). Although a minority of patients exhibited vaginal invasion or bloody discharge—potentially associated with tumor necrosis or secondary infections—these features did not significantly worsen with disease progression (41). Furthermore, tumor aggressiveness may manifest more in deep tissue infiltration or distant metastasis rather than directly in observable gynecological examination indicators (40). Therefore, while gynecological examination results provide important references for disease assessment, their ability to differentiate staging is limited. Comprehensive evaluation incorporating imaging and pathological features remains crucial.

The analysis based on FIGO staging shows that there is no statistically significant difference in ORR, CR, and PR between patients in stages I-III and stage IV. This may be related to the multi-target mechanism of action of Anlotinib Hydrochloride and its extensive regulation of the tumor microenvironment (32). Currently, there are still relatively few Phase II studies on the use of Anlotinib Hydrochloride in the treatment of CC. Although Professor Xu’s Phase II study also confirmed that Anlotinib Hydrochloride in combination with radiotherapy is effective in the treatment of CC (16), this study has further investigated and found that Anlotinib Hydrochloride also shows stable and good efficacy in the treatment of LACC across different FIGO stages. From a molecular perspective, Anlotinib Hydrochloride targets key signaling pathways such as VEGFR, PDGFR, and FGFR simultaneously (23). This not only directly inhibits tumor angiogenesis, a common feature of malignant tumor progression, but more importantly, it reshapes the immunosuppressive stromal network in the tumor microenvironment. By doing so, it disrupts the malignant synergistic interactions between tumor cells and the surrounding stromal cells (23). Inhibition of PDGFR can significantly reduce the activation of cancer-associated fibroblasts (CAFs), thereby decreasing the excessive deposition of the extracellular matrix and improving tumor tissue perfusion and drug permeability (24). Meanwhile, blocking the FGFR pathway further disrupts the paracrine signaling between tumor cells and EMT, weakening the tumor’s adaptive resistance (42). This multi-dimensional and holistic regulatory pattern allows Anlotinib Hydrochloride to effectively prevent the formation of micrometastases in stages I-III of LACC, and to reverse the established immunosuppressive microenvironment in stage IV cases (42). This may be the main reason why patients at different stages can benefit. In addition, the unique pharmacokinetic characteristics of Anlotinib hydrochloride, such as sustained and stable target occupancy and tissue distribution, ensure its long-lasting improvement effects on the tumor hypoxic microenvironment, this mechanism enables it to exert significant synergistic effects in both I-III and IV LACC (43). Administration of Anlotinib hydrochloride before radiotherapy can reverse tumor hypoxia in advance and increase radiosensitivity (42). Continuous administration after radiotherapy can inhibit the regenerative angiogenesis of residual tumor cells. For both I-III stage and IV stage patients, the ORR of Anlotinib combined with radiotherapy remains stable in the range of 82%-85%, which is significantly better than the stage-dependent efficacy of the Sunitinib combination regimen in historical studies (44). Compared with other TKI drugs, Anlotinib hydrochloride, while maintaining the same anti-angiogenic efficacy, has a significantly lower incidence and severity of dose-limiting toxicity (42). This superior safety feature makes long-term maintenance therapy possible and is also the key factor for maintaining stable efficacy in patients with advanced high tumor burden (16). These findings not only provide a new treatment strategy for the entire course of cervical cancer management but also suggest that in the treatment of solid tumors, multi-target intervention targeting the tumor ecosystem may become an important direction for breaking through the traditional stage-dependent efficacy differences.

Compared with previous studies that only focused on the overall efficacy of radiotherapy combined with chemotherapy, this study, for the first time, revealed through stage-stratified analysis that the multi-target anti-angiogenic effects of Anlotinib hydrochloride may enhance the sensitivity of Stage I-III tumors to radiotherapy by improving the tumor microenvironment. This study found that patients with stage I-III disease who were free from diabetes and had lower levels of CA125 and CA153 exhibited superior PFS. This may be attributed to diabetes-related metabolic dysregulation and the tumor burden/aggressiveness reflected by CA125/CA153 levels—factors more susceptible to modulation by anlotinib’s multi-target effects in early-stage disease (42). From the perspective of molecular pathophysiology, there is a complex interplay between diabetes-related metabolic disorders and the tumor microenvironment: The hyperglycemic state can promote tumor angiogenesis by activating the VEGF/VEGFR signaling pathway while inducing the upregulation of PDGFR and FGFR pathways, thereby forming a vicious cycle that promotes tumor growth (35). Anlotinib’s multi-target inhibitory action is precisely targeted at this pathological network. Especially in the early stages of the disease, when the tumor microenvironment has not yet formed a highly heterogeneous complex system, it can effectively inhibit the formation of new blood vessels and significantly improve diabetes-related metabolic microenvironmental abnormalities by simultaneously blocking key receptor tyrosine kinases such as VEGFR-2/3, PDGFR-α/β, and FGFR1-3 (45). CA125/CA153, as important biomarkers reflecting tumor burden and biological invasiveness, further confirm Anlotinib’s dual regulatory role in early-stage patients—targeting tumor cells themselves and improving the metabolic microenvironment (42). In contrast, in Stage IV patients, due to the formation of extensive metastatic foci and highly heterogeneous tumor ecosystems, single vascular-targeted therapy cannot comprehensively cover the specific microenvironmental characteristics of each metastatic focus, which explains the limited PFS benefit (7). This study innovatively used a stage-stratified analysis method, not only confirming the stage-dependent efficacy of Anlotinib in breast cancer treatment but more importantly revealing the dynamic association between metabolic disorders, tumor microenvironment, and targeted treatment response. This provides a theoretical basis for precision stratified treatment strategies and makes up for the limitations of existing studies that focus on overall efficacy while neglecting stage differences.

This study still has some limitations. First, as a single-arm, single-center Phase II exploratory study, the relatively limited sample size may affect the universality and reliability of the results. Further validation through multicenter randomized controlled trials will be needed in the future. Second, the study did not delve into the specific molecular mechanisms of the synergistic effect between Anlotinib Hydrochloride and radiotherapy, especially the impact on immune regulation and metabolic reprogramming in the tumor microenvironment. This limits a comprehensive understanding of its therapeutic potential.

## 5 Conclusion

In summary, this study provides preliminary evidence for the efficacy and safety of anlotinib hydrochloride combined with radiotherapy in the treatment of locally advanced cervical cancer. Although the findings indicate that comorbidities such as diabetes and hypertension require particular attention and active management to optimize safety under this regimen, the results nonetheless offer a new therapeutic option for clinical practice. Despite the limitations of sample size and mechanistic exploration, its multi-target synergistic effect and good tolerability lay an important foundation for future studies. Further optimization of the treatment regimen and clarification of the beneficiary population will be needed through larger-scale Phase III trials and translational research in the future.

## Data Availability

The original contributions presented in the study are included in the article/**Supplementary Material**. Further inquiries can be directed to the corresponding authors.

## Supplementary material

Supplementary Table 1 Physical examination findings (N = 62).

Supplementary Table 2 Complete blood count results (N = 62). LY%: Lymphocyte percentage. NEUT%: Neutrophil percentage. WBC, White blood cell count; PLT, Platelet count; RBC, Red blood cell count; FBG, Fasting blood glucose; CEA, Carcinoembryonic antigen; CA724, Carbohydrate antigen 724; AFP, Alpha-fetoprotein; CA199, Carbohydrate antigen 199; CA125, Carbohydrate antigen 125; Cyfra21.1, Cytokeratin 19 fragment 21.1; CA153, Carbohydrate antigen 153; SCC, Squamous cell carcinoma antigen.

Supplementary Table 3 Baseline characteristics of patients by disease stage.

Supplementary Table 4 Physical examination findings of patients by disease stage.

Supplementary Table 5 Cervical cancer status of patients by disease stage.

Supplementary Table 6 Complete blood count results of patients by disease stage.

Supplementary Table 7 Adverse events in the patients by disease stage.

Supplementary Table 8 Efficacy measures per RECIST v1.1 guidelines of patients by disease stage.

Supplementary Table 9 Univariate Cox proportional hazards regression analyses of PFS in stage I-III patients.

Supplementary Table 10 Univariate Cox proportional hazards regression analyses of PFS in stage IV patients.

Supplementary Figure 1 Trial profile.

Supplementary Figure 2 Forest plot of PFS from Cox regression analysis stratified by disease stage. **(A)** Forest plot of I-III patients. **(B)** Forest plot of IV patients.
