# Supplementary material for: Efficacy and safety of anlotinib hydrochloride combined with concurrent radiotherapy in the treatment of locally advanced cervical cancer: a single-arm, single-center, exploratory, phase II clinical study
Source: Front Oncol. 2025 Nov 20;15:1662160. doi: 10.3389/fonc.2025.1662160 (PMC12676224; doi:10.3389/fonc.2025.1662160)
Supplement: Supplementary Table 1 — Physical examination findings (N = 62). [file Table1.docx]

| Characteristic | Patients |
| --- | --- |
| Vaginal discharge amount |  |
| Scanty | 47 (75.81) |
| Moderate | 12 (19.35) |
| Copious | 3 (4.84) |
| Vaginal discharge color |  |
| White | 40 (64.52) |
| Purulent yellow | 11 (17.74) |
| Blood-tinged | 11 (17.74) |
| Presence of odor |  |
| Yes | 14 (22.58) |
| No | 48 (77.42) |
| Vaginal involvement |  |
| Yes | 5 (8.06) |
| No | 57 (91.94) |

**Table 1 Physical examination findings (N=62)**
