# Supplementary material for: Efficacy and safety of anlotinib hydrochloride combined with concurrent radiotherapy in the treatment of locally advanced cervical cancer: a single-arm, single-center, exploratory, phase II clinical study
Source: Front Oncol. 2025 Nov 20;15:1662160. doi: 10.3389/fonc.2025.1662160 (PMC12676224; doi:10.3389/fonc.2025.1662160)
Supplement: Supplementary Table 2 — Complete blood count results (N = 62). LY%: Lymphocyte percentage. NEUT%: Neutrophil percentage. WBC, White blood cell count; PLT, Platelet count; RBC, Red blood cell count; FBG, Fasting blood glucose; CEA, Carcinoembryonic antigen; CA724, Carbohydrate antigen 724; AFP, Alpha-fetoprotein; CA199, Carbohydrate antigen 199; CA125, Carbohydrate antigen 125; Cyfra21.1, Cytokeratin 19 fragment 21.1; CA153, Carbohydrate antigen 153; SCC, Squamous cell carcinoma antigen. [file Table2.docx]

**Table 2 Complete blood count results (N=62)**

| Characteristic | Patients |
| --- | --- |
| LY%, % | 21.72±8.92 |
| NEUT%, % | 63.66±12.18 |
| WBC, 10^9^/L | 5.14 (3.91, 7.25) |
| PLT, 10^9^/L | 228 (166.75, 292.50) |
| RBC, 10^12^/L | 3.60 (3.27, 4.12) |
| FBG, mmol/L | 4.77 (4.40, 5.13) |
| CEA, ng/mL | 1.78 (1.09, 3.33) |
| CA724, U/mL | 1.23 (0.82, 2.13) |
| AFP, ng/mL | 1.76 (1.46, 2.43) |
| CA199, U/mL | 7.35 (4.34, 13.00) |
| CA125, U/mL | 14.90 (8.40, 29.15) |
| Cyfra21.1, ng/mL | 1.90 (1.19, 3.63) |
| CA153, U/mL | 13.30 (7.53, 20.25) |
| SCC, ng/mL | 1.18 (0.80, 5.78) |

LY%: Lymphocyte percentage. NEUT%: Neutrophil percentage. WBC: White blood cell count. PLT: Platelet count. RBC: Red blood cell count. FBG: Fasting blood glucose. CEA: Carcinoembryonic antigen. CA724: Carbohydrate antigen 724. AFP: Alpha-fetoprotein. CA199: Carbohydrate antigen 199. CA125: Carbohydrate antigen 125. Cyfra21.1: Cytokeratin 19 fragment 21.1. CA153: Carbohydrate antigen 153. SCC: Squamous cell carcinoma antigen.
