# Supplementary material for: Efficacy and safety of anlotinib hydrochloride combined with concurrent radiotherapy in the treatment of locally advanced cervical cancer: a single-arm, single-center, exploratory, phase II clinical study
Source: Front Oncol. 2025 Nov 20;15:1662160. doi: 10.3389/fonc.2025.1662160 (PMC12676224; doi:10.3389/fonc.2025.1662160)
Supplement: Supplementary Table 3 — Baseline characteristics of patients by disease stage. [file Table3.docx]

**Table 3 Baseline characteristics of patients by disease stage**

| Characteristic | I-III patients (n=36) | IV patients (n=17) | *t/Z/χ^2^* | *P* |
| --- | --- | --- | --- | --- |
| Age, years | 56.00 (46.00, 67.75) | 63.00 (53.50, 72.00) | -1.897 | 0.058 |
| Ethnic group |  |  | 6.915 | 0.032 |
| Han ethnic group | 9 (25.00) | 5 (29.41) |  |  |
| Uyghurs ethnic group | 19 (52.78) | 3 (17.65) |  |  |
| Kazak ethnic group | 8 (22.22) | 9 (52.94) |  |  |
| Marital status |  |  | 0.746 | 0.689 |
| Married | 27 (75.00) | 12 (70.59) |  |  |
| Single | 1 (2.78) | 0 (0.00) |  |  |
| Divorced or widowed | 8 (22.22) | 5 (29.41) |  |  |
| Duration of symptoms, months | 2.00 (1.00, 7.25) | 4.00 (2.00, 10.00) | -1.516 | 0.129 |
| Diabetes |  |  | 0.008 | 0.929 |
| Yes | 6 (16.67) | 3 (17.65) |  |  |
| No | 30 (83.33) | 14 (82.35) |  |  |
| Hypertension |  |  | 0.082 | 0.775 |
| Yes | 12 (33.33) | 5 (29.41) |  |  |
| No | 24 (66.67) | 12 (70.59) |  |  |
| History of other cancers |  |  | 1.746 | 0.186 |
| Yes | 1 (2.78) | 2 (11.76) |  |  |
| No | 35 (97.22) | 15 (88.24) |  |  |
| Age at menarche, years | 15.00 (13.00, 15.00) | 15.00 (12.50, 15.00) | -0.381 | 0.703 |
| Duration of menstruation, days | 5.00 (5.00, 5.00) | 5.00 (5.00, 5.00) | -0.035 | 0.972 |
| Menstrual cycle length, days | 28.00 (28.00, 28.00) | 28.00 (28.00, 30.00) | -0.817 | 0.414 |
| Menstrual blood loss, mL | 33.00 (29.00, 38.50) | 30.00 (28.00, 35.00) | -1.425 | 0.154 |
| Menopausal status |  |  | 0.640 | 0.424 |
| Yes | 26 (72.22) | 14 (82.35) |  |  |
| No | 10 (27.78) | 3 (17.65) |  |  |
| Gravidity, n | 5.00 (3.00, 7.00) | 5.00 (3.00, 6.00) | -0.019 | 0.985 |
| Parity, n | 3.00 (2.00, 5.00) | 3.00 (3.00, 5.00) | -1.050 | 0.294 |
| Number of abortions, n | 1.00 (0.00, 2.00) | 1.00 (0.00, 2.00) | -1.309 | 0.191 |
| Height, m | 1.58±0.06 | 1.54±0.06 | 2.247 | 0.029 |
| Weight, kg | 63.06±11.76 | 59.47±10.57 | 1.068 | 0.290 |
| BMI, kg/m^2^ | 25.03±3.92 | 25.03±4.46 | 0.000 | 1.000 |
| Smoking history |  |  | 0.622 | 0.430 |
| Yes | 19 (52.78) | 7 (41.18) |  |  |
| No | 17 (47.22) | 10 (58.82) |  |  |
| Alcohol consumption |  |  | 0.369 | 0.543 |
| Yes | 4 (11.11) | 1 (5.88) |  |  |
| No | 32 (88.89) | 16 (94.12) |  |  |
