# Supplementary material for: Efficacy and safety of anlotinib hydrochloride combined with concurrent radiotherapy in the treatment of locally advanced cervical cancer: a single-arm, single-center, exploratory, phase II clinical study
Source: Front Oncol. 2025 Nov 20;15:1662160. doi: 10.3389/fonc.2025.1662160 (PMC12676224; doi:10.3389/fonc.2025.1662160)
Supplement: Supplementary Table 4 — Physical examination findings of patients by disease stage. [file Table4.docx]

**Table 4 Physical examination findings of patients by disease stage**

| Characteristic | I-III patients (n=36) | IV patients (n=17) | *χ^2^* | *P* |
| --- | --- | --- | --- | --- |
| Vaginal discharge amount |  |  | 2.366 | 0.306 |
| Scanty | 27 (75.00) | 15 (88.24) |  |  |
| Moderate | 8 (22.22) | 1 (5.88) |  |  |
| Copious | 1 (2.78) | 1 (5.88) |  |  |
| Vaginal discharge color |  |  | 0.282 | 0.868 |
| White | 23 (63.89) | 12 (70.59) |  |  |
| Purulent yellow | 6 (16.67) | 2 (11.76) |  |  |
| Blood-tinged | 7 (19.44) | 3 (17.65) |  |  |
| Presence of odor |  |  | 0.356 | 0.550 |
| Yes | 27 (75.00) | 3 (17.65) |  |  |
| No | 9 (25.00) | 14 (82.35) |  |  |
| Vaginal involvement |  |  | 0.638 | 0.424 |
| Yes | 2 (5.56) | 2 (11.76) |  |  |
| No | 34 (94.44) | 15 (88.24) |  |  |
