# Supplementary material for: Efficacy and safety of anlotinib hydrochloride combined with concurrent radiotherapy in the treatment of locally advanced cervical cancer: a single-arm, single-center, exploratory, phase II clinical study
Source: Front Oncol. 2025 Nov 20;15:1662160. doi: 10.3389/fonc.2025.1662160 (PMC12676224; doi:10.3389/fonc.2025.1662160)
Supplement: Supplementary Table 5 — Cervical cancer status of patients by disease stage. [file Table5.docx]

**Table 5 Cervical cancer status of patients by disease stage**

| Characteristic | I-III patients (n=36) | IV patients (n=17) | *t/χ^2^* | *P* |
| --- | --- | --- | --- | --- |
| Pathological type |  |  | 0.998 | 0.318 |
| Squamous cell carcinoma | 33 (91.67) | 14 (82.35) |  |  |
| Adenocarcinoma | 3 (8.33) | 3 (17.65) |  |  |
| ECOG PS |  |  | 0.749 | 0.688 |
| 0 | 21 (58.33) | 8 (47.06) |  |  |
| 1 | 14 (38.89) | 8 (47.06) |  |  |
| 2 | 1 (2.78) | 1 (5.88) |  |  |
| Metastatic status |  |  | 1.976 | 0.160 |
| Yes | 2 (5.56) | 3 (17.65) |  |  |
| No | 34 (94.44) | 14 (82.35) |  |  |
| Number of metastatic sites |  |  | 0.638 | 0.424 |
| 0~1 | 34 (94.44) | 15 (88.24) |  |  |
| 2 | 2 (5.56) | 2 (11.76) |  |  |
| Target lesion size, cm | 4.54±0.72 | 4.45±0.75 | 0.447 | 0.657 |
