# Supplementary material for: Efficacy and safety of anlotinib hydrochloride combined with concurrent radiotherapy in the treatment of locally advanced cervical cancer: a single-arm, single-center, exploratory, phase II clinical study
Source: Front Oncol. 2025 Nov 20;15:1662160. doi: 10.3389/fonc.2025.1662160 (PMC12676224; doi:10.3389/fonc.2025.1662160)
Supplement: Supplementary Table 6 — Complete blood count results of patients by disease stage. [file Table6.docx]

**Table 6 Complete blood count results of patients by disease stage**

| Characteristic | I-III patients (n=36) | IV patients (n=17) | *t/Z* | *P* |
| --- | --- | --- | --- | --- |
| LY%, % | 22.82±9.40 | 20.84±7.92 | 0.752 | 0.456 |
| NEUT%, % | 63.79±11.73 | 67.32±9.71 | -1.078 | 0.286 |
| WBC, 10^9^/L | 5.35 (3.89, 7.25) | 5.60 (4.61, 7.79) | -0.972 | 0.331 |
| PLT, 10^9^/L | 228 (166.75, 292.50) | 256.00 (187.00, 294.00) | -0.753 | 0.452 |
| RBC, 10^12^/L | 3.60 (3.29, 3.99) | 3.90 (3.29, 3.99) | -1.745 | 0.081 |
| FBG, mmol/L | 4.80 (4.42, 5.09) | 4.90 (4.43, 5.18) | -0.048 | 0.962 |
| CEA, ng/mL | 1.48 (1.02, 2.76) | 2.21 (1.29, 4.92) | -1.782 | 0.075 |
| CA724, U/mL | 1.22 (0.78, 2.15) | 1.22 (0.83, 2.20) | -0.143 | 0.886 |
| AFP, ng/mL | 1.79 (1.45, 2.48) | 2.09 (1.49, 2.92) | -0.867 | 0.386 |
| CA199, U/mL | 7.30 (4.54, 13.00) | 9.39 (1.09, 14.47) | -0.076 | 0.939 |
| CA125, U/mL | 15.00 (8.38, 27.95) | 17.80 (7.75, 65.00) | -0.848 | 0.396 |
| Cyfra21.1, ng/mL | 1.95 (1.20, 3.68) | 1.93 (1.15, 8.42) | -0.297 | 0.790 |
| CA153, U/mL | 12.60 (7.23, 20.75) | 15.40 (11.50, 32.00) | -1.467 | 0.142 |
| SCC, ng/mL | 1.18 (0.73, 3.10) | 2.20 (0.75, 20.00) | -1.154 | 0.249 |
