# Supplementary material for: Efficacy and safety of anlotinib hydrochloride combined with concurrent radiotherapy in the treatment of locally advanced cervical cancer: a single-arm, single-center, exploratory, phase II clinical study
Source: Front Oncol. 2025 Nov 20;15:1662160. doi: 10.3389/fonc.2025.1662160 (PMC12676224; doi:10.3389/fonc.2025.1662160)
Supplement: Supplementary Table 7 — Adverse events in the patients by disease stage. [file Table7.docx]

**Table 7 Adverse events in the patients by disease stage**

| Characteristic | I-III patients (n=36) | IV patients (n=17) | *χ^2^* | *P* |
| --- | --- | --- | --- | --- |
| Hypothyroidism | 7 (19.44) | 5 (29.41) | 0.655 | 0.418 |
| Elevated AST | 4 (11.11) | 2 (11.76) | 0.005 | 0.944 |
| Hypertension | 6 (16.67) | 2 (11.76) | 0.217 | 0.642 |
| Diarrhea | 6 (16.67) | 6 (35.29) | 2.288 | 0.130 |
| Hypertriglyceridemia | 2 (5.56) | 1 (5.88) | 0.002 | 0.962 |
| Anemia | 4 (11.11) | 1 (5.88) | 0.369 | 0.543 |
| Hypercholesterolemia | 1 (2.78) | 1 (5.88) | 0.307 | 0.580 |
| Rash | 1 (2.78) | 2 (11.76) | 1.746 | 0.186 |
| Gingival swelling and pain | 2 (5.56) | 3 (17.65) | 1.976 | 0.160 |
| Oral ulcer | 3 (8.33) | 0 (0.00) | 1.502 | 0.220 |
| Fatigue | 8 (22.22) | 7 (41.18) | 2.044 | 0.153 |
| Radiation enteritis | 2 (5.56) | 1 (5.88) | 0.002 | 0.962 |
| Radiation cystitis | 3 (8.33) | 1 (5.88) | 0.099 | 0.753 |
| Irregular bleeding | 1 (2.78) | 0 (0.00) | 0.481 | 0.488 |
