# Supplementary material for: Efficacy and safety of anlotinib hydrochloride combined with concurrent radiotherapy in the treatment of locally advanced cervical cancer: a single-arm, single-center, exploratory, phase II clinical study
Source: Front Oncol. 2025 Nov 20;15:1662160. doi: 10.3389/fonc.2025.1662160 (PMC12676224; doi:10.3389/fonc.2025.1662160)
Supplement: Supplementary Table 8 — Efficacy measures per RECIST v1.1 guidelines of patients by disease stage. [file Table8.docx]

**Table 8 Efficacy measures per RECIST v1.1 guidelines of patients by disease stage**

| Overall study endpoint | I-III patients (n=36) | IV patients (n=17) | *χ^2^* | *P* |
| --- | --- | --- | --- | --- |
| Primary endpoint |  |  |  |  |
| ORR | 16 (44.44) | 6 (35.29) | 0.398 | 0.528 |
| Secondary endpoint |  |  |  |  |
| CR | 3 (8.33) | 0 (0.00) | 1.502 | 0.220 |
| PR | 13 (36.11) | 6 (35.29) | 0.003 | 0.954 |
| SD | 16 (44.44) | 6 (35.29) | 0.398 | 0.528 |
| PD | 4 (11.11) | 5 (29.41) | 2.743 | 0.098 |
| DCR | 32 (88.89) | 12 (70.59) | 2.743 | 0.098 |
