# Supplementary material for: Efficacy and safety of anlotinib hydrochloride combined with concurrent radiotherapy in the treatment of locally advanced cervical cancer: a single-arm, single-center, exploratory, phase II clinical study
Source: Front Oncol. 2025 Nov 20;15:1662160. doi: 10.3389/fonc.2025.1662160 (PMC12676224; doi:10.3389/fonc.2025.1662160)
Supplement: Supplementary Table 9 — Univariate Cox proportional hazards regression analyses of PFS in stage I-III patients. [file Table9.docx]

**Table 9 Univariate Cox proportional hazards regression analyses of PFS in stage I-III patients**

| Characteristic | Univariate | | |
| --- | --- | --- | --- |
|  | *HR* | 95%CI | *P* |
| Age, years | 1.228 | 1.070-1.410 | 0.003 |
| Ethnic group |  |  |  |
| Han ethnic group |  |  | 0.978 |
| Uyghurs ethnic group | 1.012 | 0.142-7.198 | 0.991 |
| Kazak ethnic group | 0.866 | 0.158-4.745 | 0.869 |
| Marital status |  |  |  |
| Married |  |  | 0.999 |
| Single | 0.961 | 0.193-4.777 | 0.961 |
| Divorced or widowed | 0.000 | 0.000-9.999 | 0.992 |
| Duration of symptoms, months | 0.832 | 0.613-1.129 | 0.238 |
| Diabetes | 0.444 | 0.199-0.988 | 0.047 |
| Hypertension | 0.179 | 0.063-0.513 | 0.001 |
| History of other cancers | 0.048 | 0.000-9.999 | 1.000 |
| Age at menarche, years | 1.121 | 0.627-2.004 | 0.700 |
| Duration of menstruation, days | 0.591 | 0.249-1.406 | 0.234 |
| Menstrual cycle length, days | 1.488 | 0.727-3.044 | 0.276 |
| Menstrual blood loss, mL | 1.006 | 0.905-1.119 | 0.910 |
| Menopausal status | 0.779 | 0.157-3.860 | 0.759 |
| Gravidity, n | 0.888 | 0.652-1.209 | 0.451 |
| Parity, n | 0.840 | 0.545-1.295 | 0.430 |
| Number of abortions, n | 0.947 | 0.505-1.775 | 0.864 |
| Height, m | 176.754 | 0.009-3343.158 | 0.227 |
| Weight, kg | 0.977 | 0.919-1.038 | 0.443 |
| BMI, kg/m^2^ | 0.863 | 0.710-1.051 | 0.142 |
| Smoking history | 0.633 | 0.151-2.650 | 0.531 |
| Alcohol consumption | 0.950 | 0.116-7.748 | 0.962 |
| Vaginal discharge amount |  |  |  |
| Scanty |  |  | 0.559 |
| Moderate | 0.002 | 0.000-99.999 | 0.973 |
| Copious | 33.002 | 0.000-99.999 | 0.970 |
| Vaginal discharge color |  |  |  |
| White |  |  | 0.982 |
| Purulent yellow | 85.697 | 0.000-99.999 | 0.955 |
| Blood-tinged | 99.570 | 0.000-99.999 | 0.955 |
| Presence of odor | 0.966 | 0.433-2.154 | 0.933 |
| Vaginal involvement | 4.753 | 0.011-2017.664 | 0.614 |
| Pathological type | 24.082 | 0.001-5344.583 | 0.533 |
| ECOG PS |  |  |  |
| 0 |  |  | 0.794 |
| 1 | 707.243 | 0.000-1352.768 | 0.964 |
| 2 | 114.324 | 0.000-4239.172 | 0.962 |
| Metastatic status | 4.612 | 0.001-2166.179 | 0.723 |
| Number of metastatic sites | 0.217 | 0.000-1018.463 | 0.723 |
| Target lesion size, cm | 1.523 | 0.573-4.048 | 0.399 |
| LY%, % | 1.051 | 0.974-1.135 | 0.201 |
| NEUT%, % | 0.988 | 0.928-1.052 | 0.704 |
| WBC, 10^9^/L | 0.811 | 0.554-1.187 | 0.281 |
| PLT, 10^9^/L | 0.998 | 0.992-1.005 | 0.629 |
| RBC, 10^12^/L | 0.525 | 0.210-1.316 | 0.169 |
| FBG, mmol/L | 0.755 | 0.417-1.366 | 0.353 |
| CEA, ng/mL | 1.036 | 0.991-1.082 | 0.117 |
| CA724, U/mL | 0.769 | 0.357-1.658 | 0.503 |
| AFP, ng/mL | 0.890 | 0.427-1.855 | 0.755 |
| CA199, U/mL | 0.994 | 0.943-1.048 | 0.819 |
| CA125, U/mL | 1.002 | 1.000-1.005 | 0.037 |
| Cyfra21.1, ng/mL | 1.024 | 1.000-1.049 | 0.053 |
| CA153, U/mL | 1.115 | 1.023-1.214 | 0.013 |
| SCC, ng/mL | 1.029 | 0.992-1.068 | 0.124 |
| Hypothyroidism | 0.834 | 0.374-1.860 | 0.657 |
| Elevated AST | 4.885 | 0.033-725.443 | 0.534 |
| Hypertension | 0.597 | 0.292-1.222 | 0.158 |
| Diarrhea | 0.721 | 0.322-1.618 | 0.428 |
| Hypertriglyceridemia | 4.705 | 0.008-2763.978 | 0.634 |
| Anemia | 0.898 | 0.315-2.561 | 0.840 |
| Hypercholesterolemia | 4.612 | 0.001-2166.179 | 0.723 |
| Rash | 0.438 | 0.152-1.264 | 0.127 |
| Gingival swelling and pain | 4.706 | 0.005-4203.128 | 0.655 |
| Oral ulcer | 4.819 | 0.023-1029.294 | 0.566 |
| Fatigue | 1.370 | 0.479-3.916 | 0.557 |
| Radiation enteritis | 0.678 | 0.238-1.934 | 0.468 |
| Radiation cystitis | 4.775 | 0.011-2068.807 | 0.614 |
| Irregular bleeding | 4.612 | 0.001-2166.179 | 0.723 |
