# Supplementary material for: Efficacy and safety of anlotinib hydrochloride combined with concurrent radiotherapy in the treatment of locally advanced cervical cancer: a single-arm, single-center, exploratory, phase II clinical study
Source: Front Oncol. 2025 Nov 20;15:1662160. doi: 10.3389/fonc.2025.1662160 (PMC12676224; doi:10.3389/fonc.2025.1662160)
Supplement: Supplementary Table 10 — Univariate Cox proportional hazards regression analyses of PFS in stage IV patients. [file Table10.docx]

**Table 10 Univariate Cox proportional hazards regression analyses of PFS in stage IV patients**

| Characteristic | Univariate | | |
| --- | --- | --- | --- |
|  | *HR* | 95%CI | *P* |
| Age, years | 1.119 | 1.014-1.236 | 0.026 |
| Ethnic group |  |  |  |
| Han ethnic group |  |  | 0.177 |
| Uyghurs ethnic group | 1.039 | 0.190-5.687 | 0.965 |
| Kazak ethnic group | 3.993 | 0.850-18.748 | 0.079 |
| Marital status |  |  |  |
| Married |  |  | 1.000 |
| Single | 0.000 | 0.000-9.999 | 1.000 |
| Divorced or widowed | 1.439 | 0.298-6.961 | 0.651 |
| Duration of symptoms, months | 1.003 | 0.970-1.038 | 0.853 |
| Diabetes | 0.515 | 0.256-1.037 | 0.063 |
| Hypertension | 0.392 | 0.178-0.866 | 0.021 |
| History of other cancers | 0.367 | 0.164-0.826 | 0.015 |
| Age at menarche, years | 1.222 | 0.759-1.966 | 0.409 |
| Duration of menstruation, days | 1.155 | 0.545-2.450 | 0.707 |
| Menstrual cycle length, days | 1.096 | 0.536-2.241 | 0.802 |
| Menstrual blood loss, mL | 0.875 | 0.731-1.047 | 0.145 |
| Menopausal status | 0.846 | 0.297-2.406 | 0.754 |
| Gravidity, n | 1.484 | 0.976-2.258 | 0.065 |
| Parity, n | 1.989 | 1.121-3.529 | 0.019 |
| Number of abortions, n | 1.047 | 0.607-1.805 | 0.868 |
| Height, m | 0.098 | 0.000-3562.354 | 0.665 |
| Weight, kg | 0.977 | 0.921-1.036 | 0.442 |
| BMI, kg/m^2^ | 0.959 | 0.833-1.105 | 0.566 |
| Smoking history | 1.052 | 0.524-2.115 | 0.886 |
| Alcohol consumption | 0.768 | 0.269-2.195 | 0.622 |
| Vaginal discharge amount |  |  |  |
| Scanty |  |  | 0.776 |
| Moderate | 25.013 | 0.000-99.958 | 0.692 |
| Copious | 1.027 | 0.000-19.958 | 0.998 |
| Vaginal discharge color |  |  |  |
| White |  |  | 0.926 |
| Purulent yellow | 1.518 | 0.189-12.213 | 0.695 |
| Blood-tinged | 0.000 | 0.000-24.667 | 0.982 |
| Presence of odor | 1.391 | 0.491-3.938 | 0.534 |
| Vaginal involvement | 1.001 | 0.353-2.836 | 0.998 |
| Pathological type | 28.719 | 0.027-3110.807 | 0.346 |
| ECOG PS |  |  |  |
| 0 |  |  | 0.359 |
| 1 | 0.172 | 0.015-1.923 | 0.153 |
| 2 | 0.235 | 0.023-2.441 | 0.225 |
| Metastatic status | 0.377 | 0.186-0.765 | 0.007 |
| Number of metastatic sites | 4.851 | 1.748-13.463 | 0.002 |
| Target lesion size, cm | 0.574 | 0.242-1.361 | 0.207 |
| LY%, % | 1.033 | 0.952-1.120 | 0.435 |
| NEUT%, % | 0.993 | 0.927-1.064 | 0.852 |
| WBC, 10^9^/L | 0.813 | 0.586-1.129 | 0.217 |
| PLT, 10^9^/L | 1.000 | 0.992-1.007 | 0.942 |
| RBC, 10^12^/L | 0.757 | 0.224-2.561 | 0.655 |
| FBG, mmol/L | 0.577 | 0.231-1.443 | 0.240 |
| CEA, ng/mL | 0.981 | 0.923-1.044 | 0.551 |
| CA724, U/mL | 0.491 | 0.187-1.292 | 0.150 |
| AFP, ng/mL | 0.902 | 0.564-1.444 | 0.668 |
| CA199, U/mL | 0.975 | 0.908-1.046 | 0.474 |
| CA125, U/mL | 0.976 | 0.943-1.010 | 0.169 |
| Cyfra21.1, ng/mL | 0.994 | 0.970-1.019 | 0.628 |
| CA153, U/mL | 0.992 | 0.963-1.022 | 0.589 |
| SCC, ng/mL | 0.991 | 0.955-1.029 | 0.652 |
| Hypothyroidism | 0.860 | 0.430-1.721 | 0.670 |
| Elevated AST | 1.001 | 0.353-2.836 | 0.998 |
| Hypertension | 1.094 | 0.386-3.100 | 0.866 |
| Diarrhea | 1.115 | 0.558-2.231 | 0.757 |
| Hypertriglyceridemia | 4.810 | 0.026-894.608 | 0.556 |
| Anemia | 4.810 | 0.026-894.608 | 0.556 |
| Hypercholesterolemia | 1.000 | 0.016-64.388 | 1.000 |
| Rash | 5.213 | 0.115-236.933 | 0.396 |
| Gingival swelling and pain | 0.903 | 0.411-1.985 | 0.799 |
| Oral ulcer | 1.000 | 0.000-999.999 | 1.000 |
| Fatigue | 0.993 | 0.512-1.925 | 0.983 |
| Radiation enteritis | 4.810 | 0.026-894.608 | 0.556 |
| Radiation cystitis | 0.452 | 0.146-1.400 | 0.169 |
| Irregular bleeding | 1.000 | 0.000-999.999 | 1.000 |
