# Supplementary figures and images for: Efficacy and safety of anlotinib hydrochloride combined with concurrent radiotherapy in the treatment of locally advanced cervical cancer: a single-arm, single-center, exploratory, phase II clinical study
Source: Front Oncol. 2025 Nov 20;15:1662160. doi: 10.3389/fonc.2025.1662160 (PMC12676224; doi:10.3389/fonc.2025.1662160)

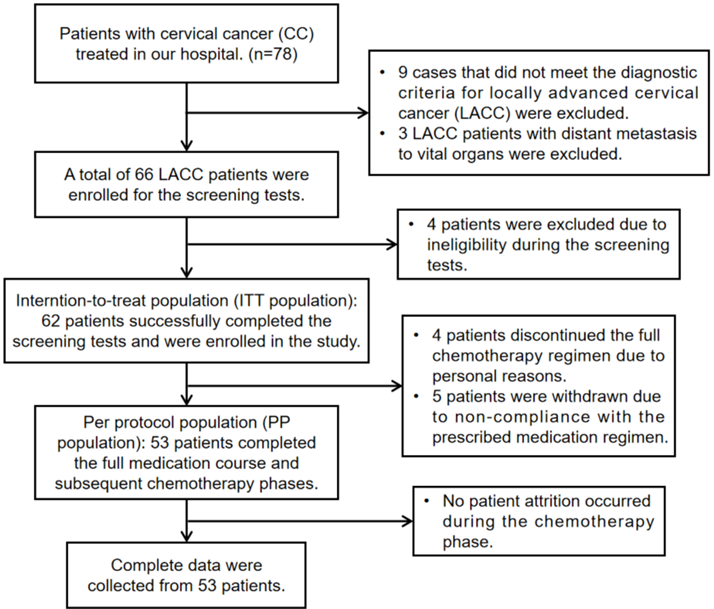


**Figure 1 Trial profile.**

Supplement: Supplementary Figure 1 — Trial profile. [file DataSheet1.docx]

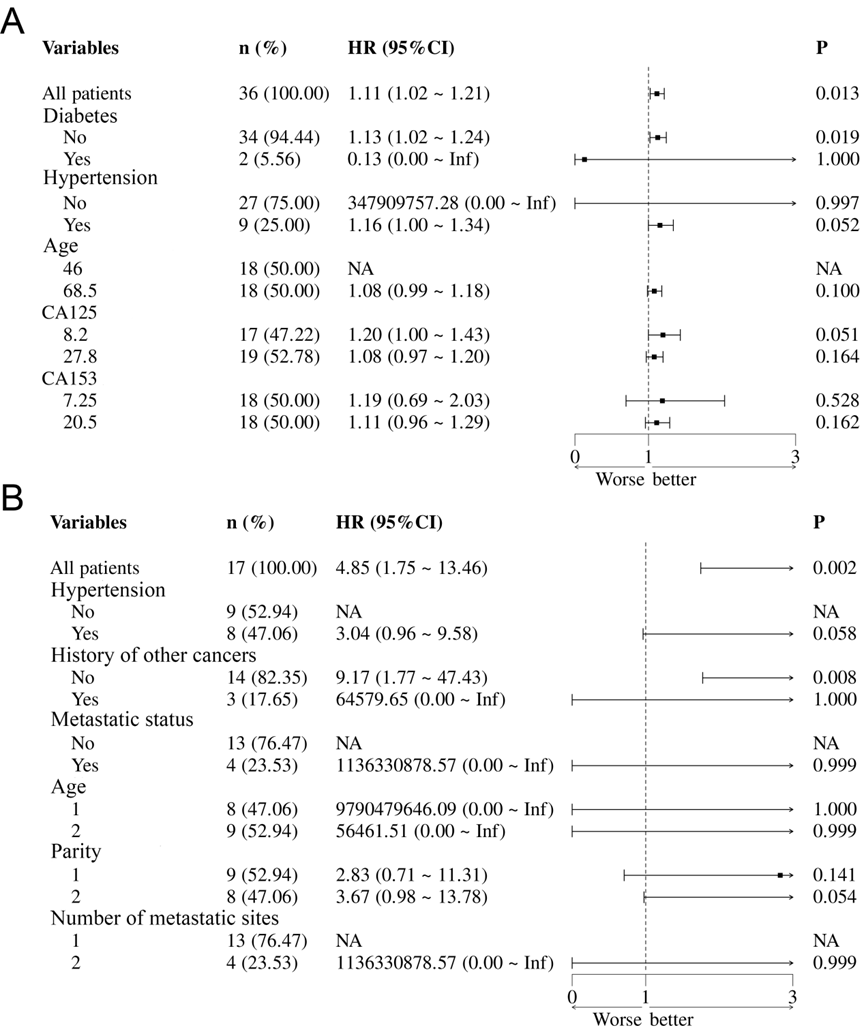


**Figure 5 Forest plot of PFS from Cox regression analysis stratified by disease stage**

Supplement: Supplementary Figure 2 — Forest plot of PFS from Cox regression analysis stratified by disease stage. (A) Forest plot of I-III patients. (B) Forest plot of IV patients. [file DataSheet2.docx]
